# Expanding our understanding of synucleinopathies: proteinopathy, proteinopenia, and lipidopathy

**DOI:** 10.1111/febs.70011

**Published:** 2025-02-25

**Authors:** Manuel Flores‐León, Tiago F. Outeiro

**Affiliations:** ^1^ Department of Experimental Neurodegeneration, Center for Biostructural Imaging of Neurodegeneration University Medical Center Göttingen Germany; ^2^ Translational and Clinical Research Institute, Faculty of Medical Sciences Newcastle University UK; ^3^ Max Planck Institute for Multidisciplinary Sciences Göttingen Germany; ^4^ Deutsches Zentrum für Neurodegenerative Erkrankungen (DZNE) Göttingen Germany

**Keywords:** alpha‐synuclein, lipids, Parkinson's disease, protein aggregation, synucleinopathy, transcription

## Abstract

A possible consequence of the process of protein aggregation in neurodegenerative diseases is the depletion of soluble protein species (proteinopenia), which may, at least in some cases, reduce protein function/activity. This concept, which is often overlooked, may play a role in synucleinopathies such as Parkinson's disease (PD), and dementia with Lewy bodies (DLB), where the protein α‐synuclein (aSyn) is known to accumulate in insoluble inclusions. aSyn is at the crossroads between cellular proteostasis and lipidostasis networks and, therefore, we must be aware of the complexity we face when we try to understand the molecular basis of synucleinopathies. Importantly, aSyn and β‐glucocerebrosidase (GCase), a sphingolipid hydrolase also strongly implicated in PD and DLB, are connected to lipid biology and to protein quality control function. Thus, changes in the normal relationship between these two proteins may shift the balance in the cell and lead to proteinopathy and/or proteinopenia, while also affecting lipidostasis of cells in the brain. Thus, pathological mechanisms that are a consequence of (a) loss‐of‐function, (b) gain‐of‐toxic function, and (c) alterations in lipidostasis need to be carefully analyzed and integrated in our study of the molecular underpinnings of neurodegenerative mechanisms. Here, we highlight implications of the depletion of the soluble form of aSyn, and of GCase, and discuss how state‐of‐the‐art ‘omics technologies’ could be deployed to assist in the clinical assessment of synucleinopathies.

## Graphical abstract

Neurodegeneration is thought to arise due to the disruption of the protein homeostasis. In synucleinopathies, alterations in the levels of functional and aggregated αSyn lead to proteinopenia and proteinopathy, respectively. αSyn and β‐glucocerebrosidase are closely linked to lipid metabolism, impacting organelle function and lipidostasis. Emerging ‘omic’ technologies offer powerful approaches for unraveling the complex molecular interactions driving these conditions, providing deeper insights into the pathophysiology of synucleinopathies, and paving the way for novel therapeutic strategies.

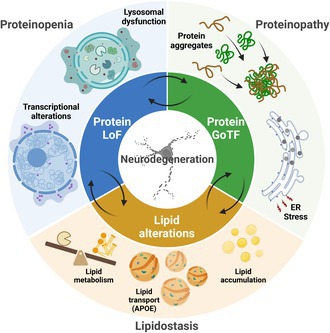

**Abbreviations**
ApoE: apolipoprotein E
aSyn: α‐synuclein
CBE: conduritol‐β‐epoxide
CMA: chaperon‐mediated autophagy
DETs: differentially expressed transcripts
DLB: dementia with Lewy bodies
ER: endoplasmic reticulum
GCase: β‐glucocerebrosidase
GoT: gain‐of‐toxic function
GWAS: genome wide association studies
KO: knock‐out
LB: Lewy bodies
LLPS: liquid–liquid phase separation
LN: Lewy neurites
LoF: loss‐of‐function
LRRK2: leucine‐rich repeat kinase 2
LUHMES: Lund human mesencephalic neuronal cell line
PD: Parkinson's disease
PRS: polygenic risk score
RNAseq: RNA sequencing
TDP‐43: transactive response DNA binding protein of 43 kDa
TWAS: transcriptomic‐wide association studies
UPR: unfolded protein response
VLDL: very low‐density lipoprotein

## Introduction

Neurodegenerative diseases, such as Parkinson's disease (PD), and dementia with Lewy bodies (DLB), are known as synucleinopathies due to the accumulation of the protein α‐synuclein (aSyn) in intraneuronal protein aggregates known as Lewy bodies (LB) and Lewy neurites (LN) [1, 2, 3]. However, the precise role of these protein aggregates in neurodegeneration is still elusive. In addition to aSyn, other proteins have emerged as important players in PD, such as the Leucine‐rich repeat kinase 2 (LRRK2) [4, 5], β‐glucocerebrosidase (GCase) [6, 7] and, more recently, the transactive response DNA binding protein of 43 kDa (TDP‐43) [8, 9]. One of the consequences of protein aggregation, which is often overlooked, is the depletion of soluble (and likely functional) species of the same protein, leading to proteinopenia [10, 11, 12].

Recently, LBs were shown to have a complex composition that includes proteins, lipids, fragmented organelles, and nucleic acids [3, 13, 14, 15, 16], and although more evidence is needed regarding the role of these biomolecules and specificity in PD mechanisms, it is likely that nucleic acids and lipids are also important players in the processes that lead to neurodegeneration. Proteins like aSyn and GCase are connected to lipid biology and to protein quality control function. Even though a mechanistic link has been established between them, individual changes in the quantity of these proteins may shift the balance in the cell and lead to proteinopathy and/or proteinopenia, while also affecting lipidostasis of different cells in the brain.

Protein aggregation can lead to a gain‐of‐toxic function (GoF) of the protein aggregates, and protein depletion due to aggregation can lead to a loss‐of‐function (LoF) of the soluble species. Protein aggregation and changes in proteostasis are hallmarks of normal aging [17, 18]. Interestingly, there are cases where neurologically ‘normal’ patients show an accumulation of protein aggregates in the brain [19, 20]. This opens the possibility to explore other mechanisms such as proteinopenia rather than only focusing on the predominant view of GoF and proteinopathy and its role in pathological mechanisms that may lead to neurodegeneration.

Thus, for our understanding of the molecular underpinnings of neurodegeneration, it is essential to (a) explore how proteinopenia affects neuronal homeostasis and brain function, (b) address the possibility that, in the long run, the balance between LoF and GoF may play a role in neurodegeneration, and (c) determine the implications of these processes on lipidostasis. In the present review, we highlight the individual implications of the depletion of the soluble form of the major component of pathological protein inclusions, aSyn, and the important genetic risk factor GCase. Additionally, we discuss how ‘omics’ technologies may be integrated to characterize the molecular fingerprints of disease and help in the clinical assessment in synucleinopathies.

## Implications of aSyn depletion in neuronal survival and neuroinflammation

aSyn is a 140 amino acid protein encoded by the *SNCA* gene and is highly abundant in the brain [21, 22, 23, 24]. aSyn comprises an N‐terminal domain that adopts α‐helical structure upon interactions with membranes, a hydrophobic middle region, and a disordered C‐terminal domain [25, 26, 27]. aSyn has been reported to exist in different states, ranging from monomers to tetramers, oligomers, protofibrils, and fibrils [28, 29]. Monomers are assumed to be the most common form found in the presynapses and in the nucleus [30, 31], while oligomers and fibrils are thought to be associated with pathological states of the protein [28, 29]. Presumably, the normal aSyn function is lost from the moment when the protein changes its conformation and starts to polymerize [11]. Although the levels of aSyn oligomers and aggregates may increase during normal aging, and in neurodegenerative diseases [32, 33], these assemblies, in particular the oligomers, are thought to be dynamic, and in equilibrium with aSyn monomers.

Although the physiological function(s) of aSyn continue under research, it has been mainly characterized as a key player in neurotransmitter release (vesicle trafficking and recycling) and, in some cases, regulating the expression of dopamine‐synthesis related genes maybe through histone binding, or by activating nuclear receptors [34, 35, 36, 37].

Silencing aSyn expression in experimental models has shown, as expected, that the protein plays a role neuronal physiology. In a study performed with two shRNAs that modulated aSyn expression at different levels in the adult rodent midbrain caused degeneration of the nigral neurons [38]. The degree of neurodegeneration of each shRNA was tightly associated with their capability to downregulate aSyn. Furthermore, this neuronal loss could be prevented if endogenous rat aSyn is supplemented to the neurons [38]. In a similar study, shRNAs targeting aSyn were injected in the *substantia nigra* of nonhuman primates (St.Kitts green monkeys). After 3 months, aSyn downregulation reproduced, in region‐specific and titer‐related manner, the degeneration of tyrosine hydroxylase (TH) neurons seen in the study with rats. Interestingly, the observed pattern of nigrostriatal degeneration of the nonhuman primates was similar to the one found in PD patients [39]. Other studies associated the presence of aSyn as a modulator of gene expression. Here, CRISPR‐Cas9 was used to delete aSyn in the Lund Human Mesencephalic (LUHMES) neuronal cell line to evaluate altered physiological cellular functions that might be associated with neuronal activity and ultimately neurodegeneration. This knock‐out (KO) besides leading to a decrease in the expression of cell cycle and differentiation genes, it also shows a downregulation of genes associated with synaptic activity and mitochondria‐mediated apoptosis [40]. This highlights the role of the presence of aSyn for neuronal function and survival [38, 39]. Overall, these findings suggest that the neuronal toxicity observed may be associated with the deletion of aSyn through different pathways that need further confirmation and experimentation.

Aging studies demonstrated that the levels of aSyn remain unchanged but its phosphorylation in serine 129 is increased affecting processes, such as dopamine uptake [41]. Furthermore, there is a decrease in the locomotor skills and anxiety‐like behavior when analyzing and comparing the results of an open field test of 18‐ and 4‐month‐old *SNCA*−/− mice [42]. Additionally, when comparing the TH+ neurons in the *substantia nigra* of these aged mice, there is a tendency of a diminished content, associated with dopaminergic loss. Also, there is an inflammatory response, shown by an increase in GFAP, Iba1, and IL‐1β [42]. This suggests that molecular mechanisms underlying the pathology are not completely related to aSyn increased levels as it has been strongly established.

Intriguingly, overexpression of aSyn in the SH‐SY5Y cell line, even to what might be considered pathological levels, leads to improved viability and proliferation [43]. Additionally, several studies revealed an association of increased levels of aSyn with different types of cancer, like hepatomas and melanoma [44, 45, 46, 47], suggesting a role in proliferation. In general, the findings with KO or overexpression of aSyn suggest that the balance in the levels of soluble aSyn protein is important in cell survival, although the precise mechanisms involved are still unclear.

One of the proposed mechanisms that might be behind these effects is the activation of inflammatory pathways, as nigrostriatal neuronal KO of aSyn upregulates inflammatory components, such as the histocompatibility complex class 1, and induces the recruitment of activated microglia, finally leading to cell death [48]. This suggests that aSyn LoF, thus proteinopenia, might be associated with the initial neuroinflammatory response seen in synucleinopathies, preceding the GoF associated with aSyn aggregates in later stages of disease.

## Interaction of aSyn with lipids

**Fig. 1:**
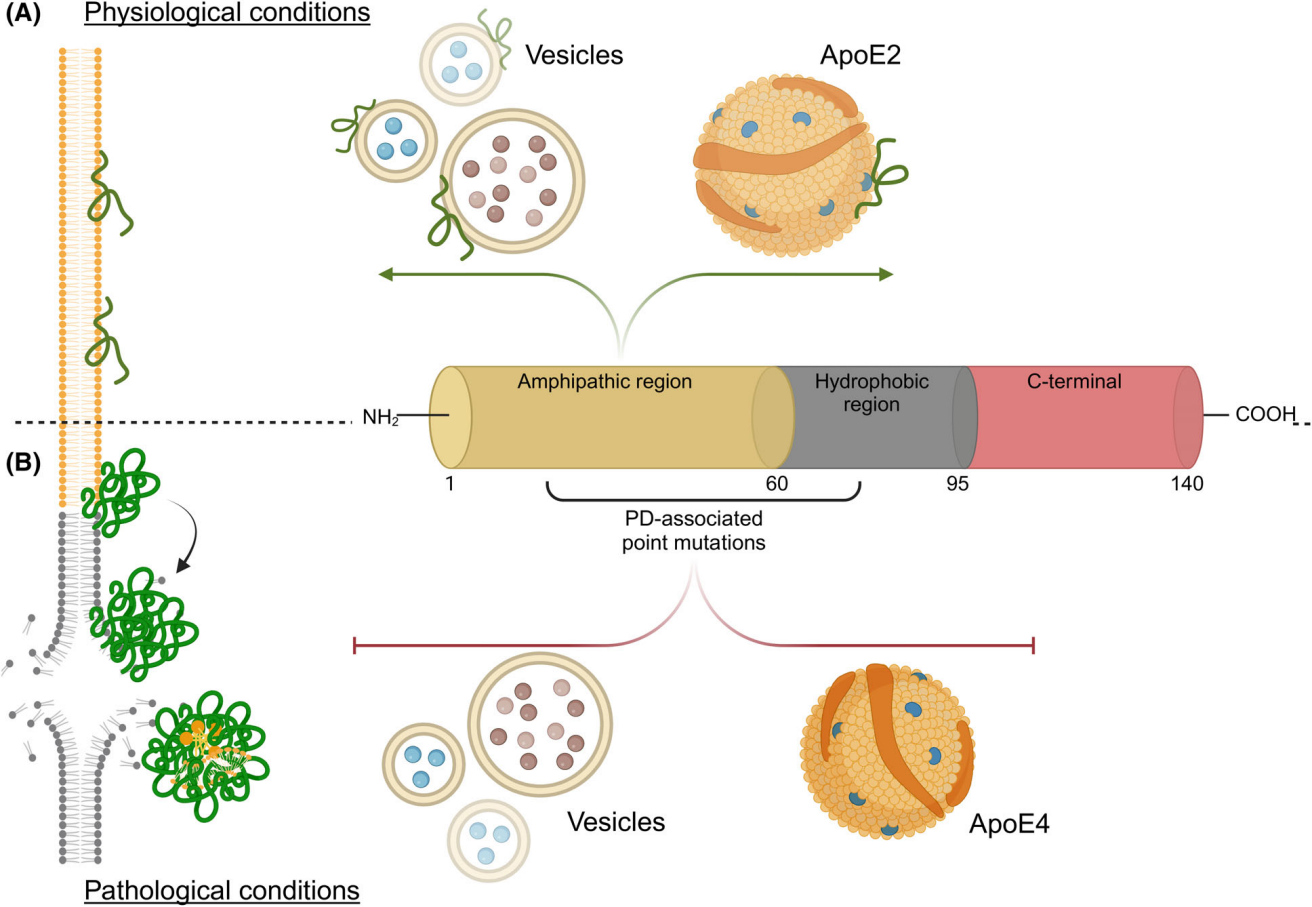
aSyn and its interactions with lipids. (A) In physiological conditions, aSyn contributes to neuronal lipidostasis through the N‐terminal amphipathic region. This domain interacts with the membranes of vesicles containing neurotransmitters, with organelle membranes rich in specific lipid species, and even with apolipoproteins, such as ApoE2. (B) In pathological conditions where lipidostasis might be disrupted, or in the presence of aSyn mutations, the interaction of aSyn with vesicles and apolipoproteins is compromised due to changes in its structure. This raises the pool of soluble aSyn, increasing the chance that it might interact with other lipids in the membranes, and also favoring nucleation, aggregation, and eventually the capture of lipid species and membrane fragments in aggregates that may, ultimately lead to the formation of Lewy bodies.

Given the amino acid composition of aSyn in the N‐terminal and in the middle hydrophobic regions (amphipathic region), aSyn binds glycosphingolipids that contain sulfate, phosphate, or sialic acid in membranes and in synaptic vesicles (Fig. 1A) [49, 50, 51, 52]. Interestingly, most of the PD‐associated mutations in the *SNCA* gene directly modify the properties of the amphipathic region, thereby affecting aSyn‐lipid interactions [49, 53, 54]. For example, the A53T and A30P mutants appear to have altered membrane‐binding affinities when compared to WT aSyn (Fig. 1B) [53, 55, 56, 57, 58].

Interestingly, glycosphingolipids, and specifically gangliosides, are reduced by up to 20% in PD [59], which might further contribute to reducing the interaction of aSyn with membranes, increasing the pool of soluble species and, possibly, making it easier for aSyn to aggregate [60]. Particularly, this is relevant in the context of endosomes and lysosomes where the lipid content is affected and an acidic environment is normally found [61, 62]. *In vitro* experiments, such as single‐molecule fluorescence tethered approach for probing of intermolecular interaction (TAPIN) [63] and aggregation assays using preformed seed fibrils (PFFs) [64], demonstrate that the stability of aSyn dimers is more than 3 times higher in an acidic (pH = 5–6) environment and that the rate of secondary nucleation increases. Thus, if lipid interactions and composition are altered in the endolysosomal system, where there is an acidic pH, then an increased aSyn aggregation rate might be plausible. Therefore, this mechanism might contribute to the uptake and aggregation of aSyn through an impaired endolysosomal system [65].

The N‐terminal aSyn domain can also interact with apolipoproteins, such as apolipoprotein E (ApoE). Strikingly, the *APOE4* allele has been identified as one of the strongest genetic risk factors for PD and DLB [66, 67, 68]. ApoE is involved in lipid exchange between neurons and glial cells [60, 61, 62, 63, 64] playing an important role in brain lipidostasis [69, 70, 71, 72, 73]. Even though, astrocytes are the main producers of ApoE in physiological conditions [71, 72], during inflammation and neuronal damage, microglia and neurons are also able to produce it [74, 75, 76]. *In vitro* and *in vivo* experimental models demonstrate that aSyn has a higher propensity to aggregate when the APOE4 variant is present, when compared to the APOE3 and APOE2 variants (Fig. 1) [77, 78]. Furthermore, the presence of ApoE4 exacerbates the aggregation of aSyn, alongside with an increase in astrogliosis and neuronal loss [78, 79]. Interestingly, transcriptomic profiling of a mouse model of synucleinopathy (based on the overexpression of aSyn via adeno‐associated viral injections into both lateral ventricles) carrying the ApoE4 variant shows alterations in lipid and energy metabolism pathways [79]. Conversely, this same synucleinopathy mouse model had reduced aSyn aggregation when carrying the ApoE2 variant [80], which might reflect the protective role of this allele. Furthermore, studies in human *postmortem* samples revealed that the APOE4 variant carriers had an increased LB pathology in DLB cases [81, 82], highlighting the importance of the ApoE variants in disease. This was further confirmed in another set of human samples were a high quantity of LBs contained fragments of APOE and the CSF of PD patients was enriched with APOE along with aSyn [83, 84].

Even though APOE has been established as a risk factor for synucleinopathies, there are still inconclusive studies suggesting that the different alleles are more related to the decrease of cognitive function rather than motor symptoms [85, 86, 87, 88]. Thus, APOE variants are currently proposed to be a risk factor for the progression of synucleinopathies with just LB pathology, like PD, to dementia, such as DLB or Parkinson's Disease Dementia (PDD). It is interesting to point out that APOE variants are also considered as a risk factor for other neurodegenerative diseases, such as Alzheimer's disease [89, 90, 91], and they are also related to inflammation [92, 93], a common process found in neurodegeneration. Given this and how sometimes synucleinopathies and amyloidopathies can coexist in the brain during neurodegeneration, it might be plausible to think that APOE variants are related to a more general neurodegenerative context. Nevertheless, it is important to continue the efforts in dissecting the mechanisms that are affected by APOE variants in the presence and/or absence of aSyn and in other neurodegenerative diseases.

Interestingly, the interaction of aSyn with lipids and membranes raises the question of whether this is related to the presence of lipids and shattered organelle membranes in some LBs [14, 16]. aSyn nucleation appears to start on the surface of membranes, and as the oligomers grow, some of them adopt a spherical shape on the edges (Fig. 1B) [94, 95], possibly related to a liquid–liquid phase separation (LLPS) phenomenon that was recently shown to be promoted by lipids. This mechanism might explain the complex structure of LBs, with a lipidic core derived from organelle‐membrane fragments that were captured, possibly by aSyn, into LBs [14, 16, 94]; nevertheless, further research on the specificity of these interactions and their contribution to aSyn pathology needs to be performed.

## Lysosomal and ER alterations due to changes in lipidostasis

Mutations in *GBA1*, the gene encoding for GCase, have been identified as risk variants in PD and DLB patients through genome wide association studies (GWAS) [96, 97, 98, 99, 100]. GCase is a lysosomal enzyme that regulates sphingolipid metabolism by transforming glucosylceramide into ceramide and glucose [101, 102], but the precise mechanisms by which diseases are triggered are still under research.

**Fig. 2:**
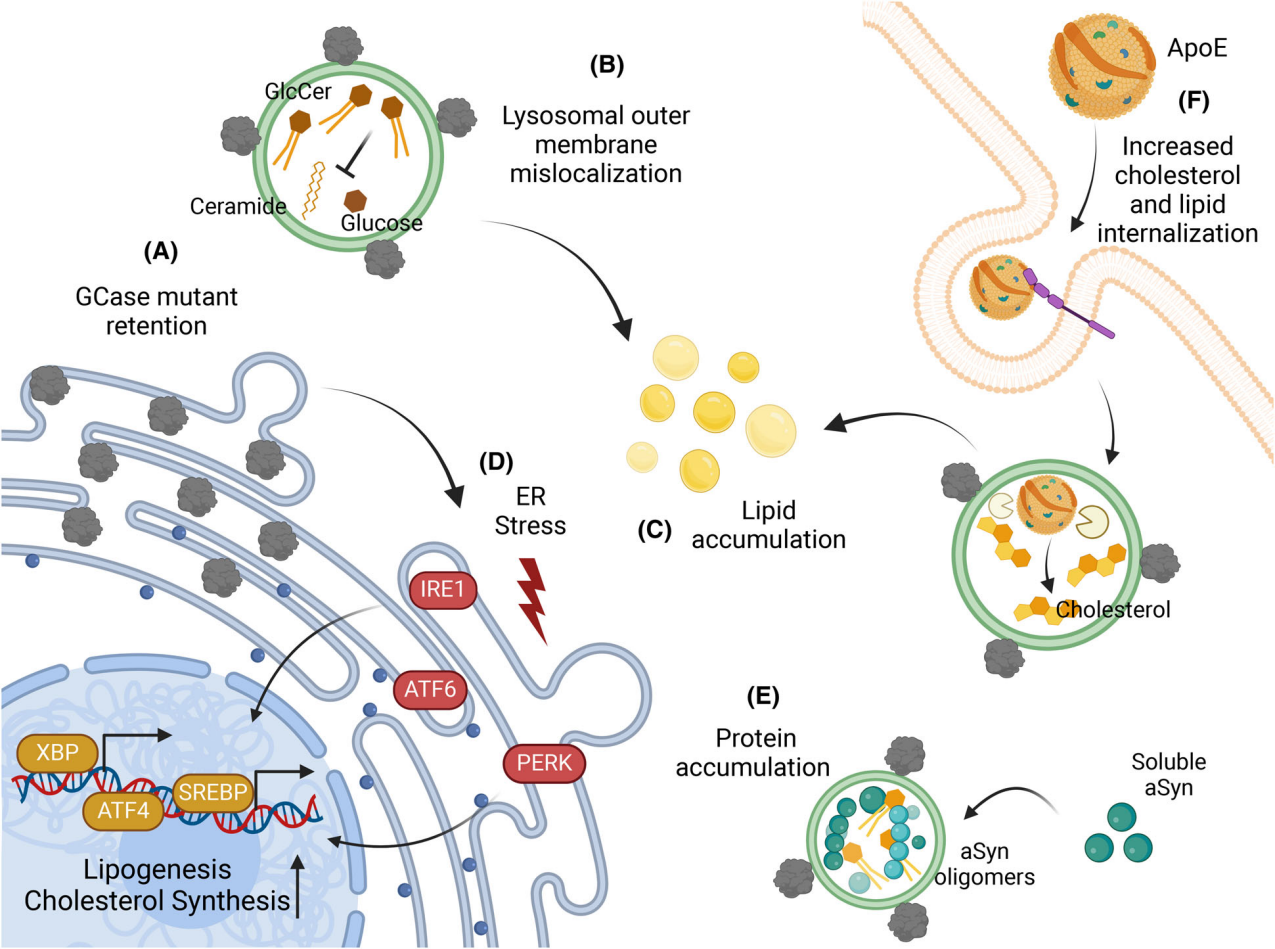
Relationship between β‐glucocerebrosidase (GCase) mutants and lipid and protein accumulation. (A) GCase mutants retained in the endoplasmic reticulum (ER) are recognized as ‘misfolded’ proteins, triggering ER stress and activating the unfolded protein response (UPR). (B) GCase mutants are not only retained in the ER, but they can be mislocalized onto the outer lysosomal membrane. This deficiency and mislocalization in lysosomes result in the accumulation of glucosylceramide (GlcCer), decreasing lysosomal activity. This is associated with lipid (C) and protein accumulation, such as aSyn (E). (D) Components of the ER stress response pathways, such as XBP and PERK, are also involved in lipogenesis and cholesterol synthesis by regulating the transcription of several genes. (E) Sphingolipid excess in lysosomes can promote the formation of aSyn oligomers, contributing to the aggregation process. (F) Lipoproteins containing ApoE can be internalized into lysosomes, where the lipids are hydrolyzed to free fatty acids and cholesterol. When GCase is not present/functional, cholesterol accumulates inside lysosomes, contributing to alterations in lipidostasis.

One of the cellular processes affected by mutations is the folding time of GCase by the resident chaperones in the endoplasmic reticulum (ER). A longer retention time in the ER signals a stress response [103, 104, 105], and this activates de Unfolded Protein Response (UPR) that, ultimately, leads to a decrease in the levels of GCase in neuronal lysosomes (Fig. 2A) [106, 107, 108]. This can be straight forward interpreted as a LoF mechanism, given that there is not enough enzyme to fulfill the lysosomal duties regarding sphingolipid metabolism [109, 110]. Therefore, strategies have been investigated to overcome the LoF in homozygous carriers, such as the use of pharmacological chaperones that work along with the endogenous chaperone system to aid in the folding of the mutant GCase, and delivery to lysosomes [108]. Nevertheless, results regarding the accumulation of sphingolipid species, the activation of the UPR, and the degree of lysosomal dysfunction in heterozygous conditions are still actively investigated and debated, suggesting other mechanisms may also contribute to neuronal dysfunction.

One of the hypotheses is that GCase deficiency in the lysosomes has an impact in sphingolipid metabolism, leading to the accumulation of glucosylceramide and glucosylsphingosine [111, 112, 113]. This, in turn, is associated with reduced lysosomal activity [114, 115, 116] and, therefore, with reduced degradation of proteins such as aSyn through chaperone‐mediated autophagy (CMA) [116]. Furthermore, GCase mutants are not only retained in the ER, but also adhere to the outer lysosomal membrane (Fig. 2B) [109]. This affects the translocation system of proteins to the lumen of the lysosome, further contributing to the aggregation of proteins in the cytosol and to lysosomal dysfunction.

The ER is a cellular compartment where most lipids are produced [61, 117] and, importantly, proteins involved in ER stress responses are associated with lipid homeostasis. For example, Ire1α plays an important role in the assembly of very low‐density lipoprotein (VLDL) in the ER, and reduced levels of this protein reduce the export of triglycerides, possibly leading to an intracellular increase of lipids [118]. XBP, another key player in ER stress responses, is involved in lipogenesis by regulating the transcription of several genes associated with lipid metabolism [119]. Furthermore, the overactivation of the PERK pathway is associated with an increase in the activity of the transcription factors SREBP and ATF4, upregulating genes associated with lipogenesis and cholesterol synthesis (Fig. 2D) [120, 121]. Together, these data suggest that when GCase mutants are retained for longer in the ER, elements of the ER stress responses may also affect lipid metabolism. This further supports a close relationship between proteostasis and lipidostasis (Fig. 2C), given that any fluctuation and impairment in one of these two processes will also likely impact the other [122].

Moreover, the exchange of lipids between glia and neurons is an important mechanism to reduce the accumulation of toxic lipid species [123, 124]. GCase inhibition in the mouse brain recapitulates the redistribution of neutral lipids between neurons and glia observed in PD patients [125]. Furthermore, genetic analyses and clinical evidence show that cognitive decline is accelerated in patients carrying both *GBA1* mutations and the *APOE4* variant, when compared to patients carrying only one of them [126]. Thus, it is important to consider the role that alterations in two key proteins in lipid metabolism might have for neurodegeneration.

Experiments in APOE−/− cerebral organoids showed reduced levels of GCase alongside a reduction in ceramide intermediates [78]. Interestingly, the excess of some sphingolipids, such as glucosylceramide, promotes the formation of aSyn oligomers in acidic environments such as the lysosomes (Fig. 2E) [116]. One hypothesis is that the absence of APOE impacts membrane lipid composition, leading to endolysosomal dysfunction, lipid droplet accumulation, and GCase LoF [78]. Accordingly, GCase inhibition in mice using conduritol‐β‐epoxide (CBE) elevates the levels of LAMP1. This can be further exacerbated when reducing the activity of GCase in ApoE−/− mice [127]. Additionally, ApoE content in the cortex, hippocampus, and substantia nigra is increased when GCase is inhibited [127]. These data suggest (a) that alterations in lipid species deregulate other lipid components leading to their accumulation, and (b) that lysosomal activity might increase in response to the alterations in lipidostasis (Fig. 2F), thereby affecting proteostasis.

In summary, lipid metabolism alterations and lipid accumulation compromise cellular functions and might alter interactions with lipid‐binding proteins, such as aSyn, promoting their accumulation.

## ‘Omics’ approaches to decipher the ‘puzzle’ of synucleinopathies

Currently, clinical diagnosis of PD and other synucleinopathies is achieved upon the onset of the typical features of the diseases. Diagnosing the diseases earlier will be essential for future clinical trials and for personalized medicine, but this requires the use of different levels of biological information for enabling patient stratification [128, 129]. Therefore, other novel strategies should be considered for diagnosing, for identifying patients, for following disease progression, and for defining therapeutic strategies. In this context, basic science approaches using ‘omics’ analyses are highly suited to provide insight into the molecular basis of disease and for identifying targets for therapeutic intervention [130].

Proteomics is one of the most used approaches, not only for dissecting disease mechanisms, but also for measuring molecules of interest in a wide variety of human samples. In this context, different techniques can be exploited, such as mass spectrometry, multiplex immunoassays, or proximity extension assays [131, 132, 133, 134, 135]. Although these experimental strategies are very specific at a molecular level, they are still not widely implemented due to limited access to expensive instrumentation and to technical issues that require unique expertise [132, 134]. Nevertheless, technological advances in the field of proteomics, such as tremendous increases in sensitivity, make these technologies powerful instruments for dissecting molecular mechanisms associated with neurodegeneration.

Next generation sequencing technologies have also been important approaches in the field. Genomics enabled various GWAS that resulted in the identification of genetic variants associated with low, middle, or high risk of developing the disease. Likewise, transcriptomic studies are bringing tremendous information into the molecular mechanisms of neurodegeneration, by providing unparalleled information about coding and noncoding molecules, including chromatin and RNA modifications [115, 136, 137]. In particular, the advent of single‐cell/single‐nucleus transcriptomics is providing mechanistic insight into disease etiology and may also aid in diagnosis and in delineating treatment strategies. For this, identifying differentially expressed transcripts (DETs) due to alternative splicing is also a priority. Characterization of spliced variants might shed light into cell‐autonomous pathways and into genetic interactions leading to neurodegeneration. In synucleinopathies, such studies are still scarce, but this strategy has revealed novel transcriptomic signatures in other dementias such as AD [138, 139]. In DLB, RNA sequencing (RNAseq) and single‐cell RNAseq revealed a clear alteration in transcript ratios across cell types [136]. These sorts of analyses can lead to transcriptome‐wide association studies (TWAS), which might help further interpret disease risk arising from GWAS. Although additional detailed studies are needed to support this strategy, this clearly suggests that already identified genetic variants may harbor additional secrets that reflect the complexity of the neurodegenerative landscape.

Supervised machine learning and AI‐assisted models are starting to prove all their power for predicting and assessing pathology risk and outcome. Until recently, the most common computational technique using genomic information was the polygenic risk score (PRS) [140]. This score aims to provide a predictive metric of an individual's predisposition to develop a certain pathology based on his/her genetic landscape, using the cumulative data usually found through GWAS data [141]. The main goal is to consider as much variance as possible in order to assess the risk of pathology. Nevertheless, this approach does not explain causality, meaning that it does not take into account other risk factors that may be key players in pathology. Nevertheless, owing to their sensitivity for unraveling ‘hidden’ data within a complex dataset [142], novel supervised machine learning algorithms hold great promise for integrating information from different ‘omic’ technologies in order to improve the accuracy of genomic prediction [142, 143]. However, a major problem in most of the datasets is that they focus on particular populations, primarily, white caucasians, that are not representative of the risk that certain factors might have on other populations. In this context, it is imperative that we are aware of this bias, and that we make all efforts to study diverse populations that represent the human species as a whole, to ensure that AI and supervised machine learning approaches are not leaving any population ‘behind’.

We should also keep in mind that the brain is extremely rich in lipids, and that various lipid species have been implicated in neurodegenerative conditions, including synucleinopathies [144]. Thus, lipidomics is also an important field in the study of disease‐associated mechanisms and for the development of putative therapeutic strategies. In this context, methods such as mass spectrometry and ion chromatography are extremely important for assessing lipid composition in various types of biological samples (including biopsies and body fluids) [145].

Several lipidomic studies show that various lipid species are altered in the brains of PD and DLB patients [146, 147]. Particularly, species, such as cholesterol, and sphingolipids are detected in different levels in PD patients [148, 149]. Additionally, studies performed in the cerebrospinal fluid and plasma of PD patients identified different lipidomic signatures (increased monohexosylceramides, ceramides, and decreased sphingomyelin) compared to healthy controls [150, 151]. Although lipidomic profiles of PD and DLB patients have been reported, it is still challenging to compare them due to the variability in the methods and sampling used in the different studies [152].

In summary, a combination of ‘omics’ approaches, such as the ones highlighted here, will be essential to generate molecular fingerprints that can help us uncover the biological basis of disease and, thereby, to enable precision medicine even in complex neurodegenerative diseases such as synucleinopathies.

## Concluding remarks

**Fig. 3:**
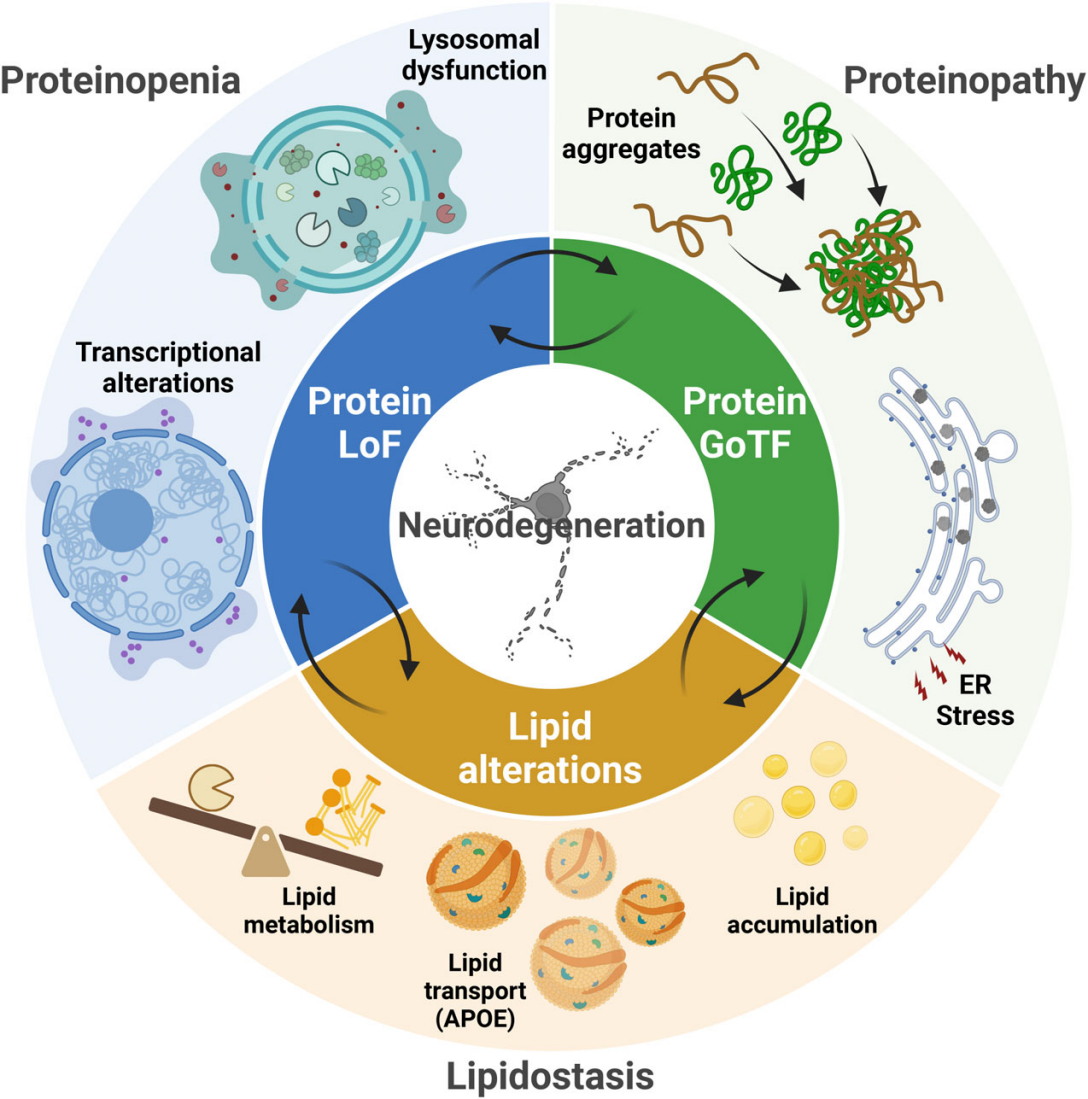
Proteinopenia, proteinopathy, and lipidostasis as key players in synucleinopathies. Different mechanisms have been implicated in neurodegenerative diseases such as synucleinopathies. Proteinopenia can lead to transcriptional alterations and lysosomal dysfunction, but at the same time these proteins can mislocalize and aggregate in the cytoplasm, or can be retained in the endoplasmic reticulum (ER), triggering the ER stress response. In turn, a reduction in the levels of functional proteins, due to mislocalization and aggregation, may lead to altered lipid metabolism, transport, and accumulation, affecting the overall neuronal lipidostasis. Most likely, all of these mechanisms coexist and contribute to disease. Thus, the interactions between loss‐of‐function (LoF), gain‐of‐toxic function (GoF), and alterations in lipidostasis need to be carefully analyzed and integrated in our quest to diagnose and treat synucleinopathies and other neurodegenerative disorders.

Synucleinopathies are highly complex, multi‐factorial, and still untreatable neurodegenerative diseases. It is a fact that, despite tremendous effort and progress in our understanding of the biology involved in synucleinopathies, successes have been very limited. Scientific advances come, often, from breaking boundaries and from challenging dogmas. In this review, we discussed the need for considering alternative, but not necessarily mutually exclusive perspectives, such as proteinopenia and lipidostasis dysfunction, in our list of synucleinopathy‐associated pathological mechanisms (Fig. 3).

Nowadays, state‐of‐the‐art ‘omics’ approaches, such as proteomics, genomics, and lipidomics, provide molecular fingerprints of cells, organs, or organisms. The challenge will be to exploit the large datasets generated by these approaches, using machine learning and AI tools, in order to understand cell physiology as a whole. This will enable us not only to formulate and test more unbiased hypothesis, but also to foster the development of biomarkers for early diagnosis, and of personalized medicine strategies for treating complex neurodegenerative diseases.

## Conflict of interest

The authors declare no conflict of interest.

## Author contributions

MF‐L designed the review outline, did the literature search, wrote the manuscript, designed, and prepared illustrations. TFO designed the review outline, performed literature research, wrote, and proofread the manuscript. All authors read and approved the final manuscript.

## References

[1] Singleton AB , Farrer M , Johnson J , Singleton A , Hague S , Kachergus J , Hulihan M , Peuralinna T , Dutra A , Nussbaum R *et al*. (2003) Alpha‐synuclein locus triplication causes Parkinson's disease. Science 302, 841. 14593171 10.1126/science.1090278

[2] Spillantini MG , Schmidt ML , Lee VMY , Trojanowski JQ , Jakes R & Goedert M (1997) α‐Synuclein in Lewy bodies. Nature 388, 839–840. 9278044 10.1038/42166

[3] Kalia LV & Lang AE (2015) Parkinson's disease. Lancet 386, 896–912. 25904081 10.1016/S0140-6736(14)61393-3

[4] Hyun CH , Yoon CY , Lee H‐J & Lee S‐J (2013) LRRK2 as a potential genetic modifier of synucleinopathies: interlacing the two major genetic factors of Parkinson's disease. Exp Neurobiol 22, 249–257. 24465140 10.5607/en.2013.22.4.249 PMC3897686

[5] O'Hara DM , Pawar G , Kalia SK & Kalia LV (2020) LRRK2 and α‐Synuclein: distinct or synergistic players in Parkinson's disease? Front Neurosci 14, 541220. 10.3389/fnins.2020.00577 PMC7311858 32625052

[6] Granek Z , Barczuk J , Siwecka N , Rozpędek‐Kamińska W , Kucharska E & Majsterek I (2023) GBA1 gene mutations in α‐synucleinopathies‐molecular mechanisms underlying pathology and their clinical significance. Int J Mol Sci 24, 2044. 36768367 10.3390/ijms24032044 PMC9917178

[7] Franco R , Sánchez‐Arias JA , Navarro G & Lanciego JL (2018) Glucocerebrosidase mutations and synucleinopathies. Potential role of sterylglucosides and relevance of studying both GBA1 and GBA2 genes. Front Neuroanat 12, 367318. 10.3389/fnana.2018.00052 PMC6031742 30002620

[8] Sampognaro PJ , Arya S , Knudsen GM , Gunderson EL , Sandoval‐Perez A , Hodul M , Bowles K , Craik CS , Jacobson MP & Kao AW (2023) Mutations in α‐synuclein, TDP‐43 and tau prolong protein half‐life through diminished degradation by lysosomal proteases. Mol Neurodegener 18, 1–22. 37131250 10.1186/s13024-023-00621-8 PMC10155372

[9] Tian T , Huang C , Tong J , Yang M , Zhou H & Xia XG (2011) TDP‐43 potentiates alpha‐synuclein toxicity to dopaminergic neurons in transgenic mice. Int J Biol Sci 7, 234–243. 21448284 10.7150/ijbs.7.234 PMC3053535

[10] Espay AJ & Lees AJ (2024) Loss of monomeric alpha‐synuclein (synucleinopenia) and the origin of Parkinson's disease. Parkinsonism Relat Disord 122, 106077. 38461037 10.1016/j.parkreldis.2024.106077

[11] Espay AJ & Okun MS (2023) Abandoning the proteinopathy paradigm in Parkinson disease. JAMA Neurol 80, 123–124. 36441542 10.1001/jamaneurol.2022.4193

[12] Ezzat K , Sturchio A & Espay AJ (2023) The shift to a proteinopenia paradigm in neurodegeneration. Handb Clin Neurol 193, 23–32. 36803814 10.1016/B978-0-323-85555-6.00001-1

[13] Leverenz JB , Umar I , Wang Q , Montine TJ , McMillan PJ , Tsuang DW , Jin J , Pan C , Shin J , Zhu D *et al*. (2007) Proteomic identification of novel proteins in cortical Lewy bodies. Brain Pathol 17, 139–145. 17388944 10.1111/j.1750-3639.2007.00048.x PMC8095629

[14] Gai WP , Yuan HX , Li XQ , Power JTH , Blumbergs PC & Jensen PH (2000) In situ and in vitro study of colocalization and segregation of α‐synuclein, ubiquitin, and lipids in Lewy bodies. Exp Neurol 166, 324–333. 11085897 10.1006/exnr.2000.7527

[15] Araki K , Yagi N , Ikemoto Y , Yagi H , Choong CJ , Hayakawa H , Beck G , Sumi H , Fujimura H , Moriwaki T *et al*. (2015) Synchrotron FTIR micro‐spectroscopy for structural analysis of Lewy bodies in the brain of Parkinson's disease patients. Sci Rep 5, 17625. 26621077 10.1038/srep17625 PMC4664933

[16] Shahmoradian SH , Lewis AJ , Genoud C , Hench J , Moors TE , Navarro PP , Castaño‐Díez D , Schweighauser G , Graff‐Meyer A , Goldie KN *et al*. (2019) Lewy pathology in Parkinson's disease consists of crowded organelles and lipid membranes. Nat Neurosci 22, 1099–1109. 31235907 10.1038/s41593-019-0423-2

[17] Trigo D , Nadais A & da Cruz e Silva OAB (2019) Unravelling protein aggregation as an ageing related process or a neuropathological response. Ageing Res Rev 51, 67–77. 30763619 10.1016/j.arr.2019.02.001

[18] Cuanalo‐Contreras K , Schulz J , Mukherjee A , Park KW , Armijo E & Soto C (2023) Extensive accumulation of misfolded protein aggregates during natural aging and senescence. Front Aging Neurosci 14, 1090109. 36778589 10.3389/fnagi.2022.1090109 PMC9909609

[19] Knopman DS , Parisi JE , Salviati A , Floriach‐Robert M , Boeve BF , Ivnik RJ , Smith GE , Dickson DW , Johnson KA , Petersen LE *et al*. (2003) Neuropathology of cognitively normal elderly. J Neuropathol Exp Neurol 62, 1087–1095. 14656067 10.1093/jnen/62.11.1087

[20] Markesbery WR , Jicha GA , Liu H & Schmitt FA (2009) Lewy body pathology in normal elderly subjects. J Neuropathol Exp Neurol 68, 816–822. 19535990 10.1097/NEN.0b013e3181ac10a7 PMC2704264

[21] Totterdell S , Hanger D & Meredith GE (2004) The ultrastructural distribution of alpha‐synuclein‐like protein in normal mouse brain. Brain Res 1004, 61–72. 15033420 10.1016/j.brainres.2003.10.072

[22] Vivacqua G , Casini A , Vaccaro R , Fornai F , Yu S & D'Este L (2011) Different sub‐cellular localization of alpha‐synuclein in the C57BL\6J mouse's central nervous system by two novel monoclonal antibodies. J Chem Neuroanat 41, 97–110. 21172422 10.1016/j.jchemneu.2010.12.003

[23] Taguchi K , Watanabe Y , Tsujimura A & Tanaka M (2016) Brain region‐dependent differential expression of alpha‐synuclein. J Comp Neurol 524, 1236–1258. 26358191 10.1002/cne.23901

[24] Withers GS , George JM , Banker GA & Clayton DF (1997) Delayed localization of synelfin (synuclein, NACP) to presynaptic terminals in cultured rat hippocampal neurons. Brain Res Dev Brain Res 99, 87–94. 9088569 10.1016/s0165-3806(96)00210-6

[25] Bertoncini CW , Fernandez CO , Griesinger C , Jovin TM & Zweckstetter M (2005) Familial mutants of α‐synuclein with increased neurotoxicity have a destabilized conformation. J Biol Chem 280, 30649–30652. 16020550 10.1074/jbc.C500288200

[26] Bartels T , Ahlstrom LS , Leftin A , Kamp F , Haass C , Brown MF & Beyer K (2010) The N‐terminus of the intrinsically disordered protein α‐synuclein triggers membrane binding and helix folding. Biophys J 99, 2116–2124. 20923645 10.1016/j.bpj.2010.06.035 PMC3042581

[27] Xu L , Nussinov R & Ma B (2016) Coupling of the non‐amyloid‐component (NAC) domain and the KTK(E/Q)GV repeats stabilize the α‐synuclein fibrils. Eur J Med Chem 121, 841–850. 26873872 10.1016/j.ejmech.2016.01.044 PMC4960003

[28] Lashuel HA , Overk CR , Oueslati A & Masliah E (2013) The many faces of α‐synuclein: from structure and toxicity to therapeutic target. Nat Rev Neurosci 14, 38–48. 23254192 10.1038/nrn3406 PMC4295774

[29] Meade RM , Fairlie DP & Mason JM (2019) Alpha‐synuclein structure and Parkinson's disease – lessons and emerging principles. Mol Neurodegener 14, 1–14. 31331359 10.1186/s13024-019-0329-1 PMC6647174

[30] Fauvet B , Mbefo MK , Fares MB , Desobry C , Michael S , Ardah MT , Tsika E , Coune P , Prudent M , Lion N *et al*. (2012) α‐Synuclein in central nervous system and from erythrocytes, mammalian cells, and *Escherichia coli* exists predominantly as disordered monomer. J Biol Chem 287, 15345–15364. 22315227 10.1074/jbc.M111.318949 PMC3346117

[31] Maroteaux L , Campanelli JT & Scheller RH (1988) Synuclein: a neuron‐specific protein localized to the nucleus and presynaptic nerve terminal. J Neurosci 8, 2804–2815. 3411354 10.1523/JNEUROSCI.08-08-02804.1988 PMC6569395

[32] David DC (2012) Aging and the aggregating proteome. Front Genet 3, 247. 23181070 10.3389/fgene.2012.00247 PMC3501694

[33] Pras A & Nollen EAA (2021) Regulation of age‐related protein toxicity. Front Cell Dev Biol 9, 637084. 33748125 10.3389/fcell.2021.637084 PMC7973223

[34] Nemani VM , Lu W , Berge V , Nakamura K , Onoa B , Lee MK , Chaudhry FA , Nicoll RA & Edwards RH (2010) Increased expression of α‐synuclein reduces neurotransmitter release by inhibiting synaptic vesicle reclustering after endocytosis. Neuron 65, 66–79. 20152114 10.1016/j.neuron.2009.12.023 PMC3119527

[35] Kontopoulos E , Parvin JD & Feany MB (2006) α‐Synuclein acts in the nucleus to inhibit histone acetylation and promote neurotoxicity. Hum Mol Genet 15, 3012–3023. 16959795 10.1093/hmg/ddl243

[36] Burré J , Sharma M , Tsetsenis T , Buchman V , Etherton MR & Südhof TC (2010) α‐Synuclein promotes SNARE‐complex assembly in vivo and in vitro. Science 329, 1663–1667. 20798282 10.1126/science.1195227 PMC3235365

[37] Baptista MJ , O'Farrell C , Daya S , Ahmad R , Miller DW , Hardy J , Farrer MJ & Cookson MR (2003) Co‐ordinate transcriptional regulation of dopamine synthesis genes by α‐synuclein in human neuroblastoma cell lines. J Neurochem 85, 957–968. 12716427 10.1046/j.1471-4159.2003.01742.x

[38] Gorbatyuk OS , Li S , Nash K , Gorbatyuk M , Lewin AS , Sullivan LF , Mandel RJ , Chen W , Meyers C , Manfredsson FP *et al*. (2010) In vivo RNAi‐mediated α‐synuclein silencing induces nigrostriatal degeneration. Mol Ther 18, 1450–1457. 20551914 10.1038/mt.2010.115 PMC2927065

[39] Collier TJ , Redmond ED , Steece‐Collier K , Lipton JW & Manfredsson FP (2016) Is alpha‐synuclein loss‐of‐function a contributor to parkinsonian pathology? Evidence from non‐human primates. Front Neurosci 10, 180553. 10.3389/fnins.2016.00012 PMC4731516 26858591

[40] Prahl J , Pierce SE , Coetzee GA & Tyson T (2022) Alpha‐synuclein negatively controls cell proliferation in dopaminergic neurons. Mol Cell Neurosci 119, 103702. 35093507 10.1016/j.mcn.2022.103702

[41] Domenicale C , Mercatelli D , Albanese F , Novello S , Vincenzi F , Varani K & Morari M (2022) Dopamine transporter, PhosphoSerine129 α‐synuclein and α‐synuclein levels in aged LRRK2 G2019S knock‐in and knock‐out mice. Biomedicine 10, 881. 10.3390/biomedicines10040881 PMC9027615 35453631

[42] Lamontagne‐Proulx J , Coulombe K , Morissette M , Rieux M , Calon F , Di Paolo T & Soulet D (2023) Sex and age differences in a progressive synucleinopathy mouse model. Biomolecules 13, 977. 37371557 10.3390/biom13060977 PMC10296285

[43] Rodríguez‐Losada N , de la Rosa J , Larriva M , Wendelbo R , Aguirre JA , Castresana JS & Ballaz SJ (2020) Overexpression of alpha‐synuclein promotes both cell proliferation and cell toxicity in human SH‐SY5Y neuroblastoma cells. J Adv Res 23, 37–45. 32071790 10.1016/j.jare.2020.01.009 PMC7016025

[44] Shekoohi S , Rajasekaran S , Patel D , Yang S , Liu W , Huang S , Yu X & Witt SN (2021) Knocking out alpha‐synuclein in melanoma cells dysregulates cellular iron metabolism and suppresses tumor growth. Sci Rep 11, 1–15. 33664298 10.1038/s41598-021-84443-y PMC7933179

[45] Yang HM , Cheng YZ , Hou TZ , Fan JK , Gu L , Zhang JN & Zhang H (2023) Upregulation of Parkinson's disease‐associated protein alpha‐synuclein suppresses tumorigenesis via interaction with mGluR5 and gamma‐synuclein in liver cancer. Arch Biochem Biophys 744, 109698. 37487948 10.1016/j.abb.2023.109698

[46] Israeli E , Yakunin E , Zarbiv Y , Hacohen‐Solovich A , Kisos H , Loeb V , Lichtenstein M , Ben‐Gedalya T , Sabag O , Pikarsky E *et al*. (2011) α‐Synuclein expression selectively affects tumorigenesis in mice modeling Parkinson's disease. PLoS One 6, e19622. 21611169 10.1371/journal.pone.0019622 PMC3097187

[47] Zanotti LC , Malizia F , Cesatti Laluce N , Avila A , Mamberto M , Anselmino LE & Menacho‐Márquez M (2023) Synuclein proteins in cancer development and progression. Biomolecules 13, 980. 37371560 10.3390/biom13060980 PMC10296229

[48] Benskey MJ , Sellnow RC , Sandoval IM , Sortwell CE , Lipton JW & Manfredsson FP (2018) Silencing alpha synuclein in mature nigral neurons results in rapid neuroinflammation and subsequent toxicity. Front Mol Neurosci 11, 36. 29497361 10.3389/fnmol.2018.00036 PMC5819572

[49] Jo E , Fuller N , Rand RP , St George‐Hyslop P & Fraser PE (2002) Defective membrane interactions of familial Parkinson's disease mutant A30P α‐synuclein. J Mol Biol 315, 799–807. 11812148 10.1006/jmbi.2001.5269

[50] Fusco G , De Simone A , Gopinath T , Vostrikov V , Vendruscolo M , Dobson CM & Veglia G (2014) Direct observation of the three regions in α‐synuclein that determine its membrane‐bound behaviour. Nat Commun 5, 3827. 24871041 10.1038/ncomms4827 PMC4046108

[51] Eliezer D , Kutluay E , Bussell R & Browne G (2001) Conformational properties of α‐synuclein in its free and lipid‐associated states. J Mol Biol 307, 1061–1073. 11286556 10.1006/jmbi.2001.4538

[52] Ferreon ACM , Gambin Y , Lemke EA & Deniz AA (2009) Interplay of α‐synuclein binding and conformational switching probed by single‐molecule fluorescence. Proc Natl Acad Sci USA 106, 5645–5650. 19293380 10.1073/pnas.0809232106 PMC2667048

[53] Perrin RJ , Woods WS , Clayton DF & George JM (2000) Interaction of human α‐synuclein and Parkinson's disease variants with phospholipids: structural analysis using site‐directed mutagenesis. J Biol Chem 275, 34393–34398. 10952980 10.1074/jbc.M004851200

[54] Candelise N , Schmitz M , Thüne K , Cramm M , Rabano A , Zafar S , Stoops E , Vanderstichele H , Villar‐Pique A , Llorens F *et al*. (2020) Effect of the micro‐environment on α‐synuclein conversion and implication in seeded conversion assays. Transl Neurodegener 9, 1–16. 31988747 10.1186/s40035-019-0181-9 PMC6966864

[55] Ferreon ACM & Deniz AA (2007) α‐Synuclein multistate folding thermodynamics: implications for protein misfolding and aggregation. Biochemistry 46, 4499–4509. 17378587 10.1021/bi602461y

[56] Robotta M , Cattani J , Martins JC , Subramaniam V & Drescher M (2017) Alpha‐synuclein disease mutations are structurally defective and locally affect membrane binding. J Am Chem Soc 139, 4254–4257. 28298083 10.1021/jacs.6b05335

[57] Jensen PH , Nielsen MS , Jakes R , Dotti CG & Goedert M (1998) Binding of α‐synuclein to brain vesicles is abolished by familial Parkinson's disease mutation. J Biol Chem 273, 26292–26294. 9756856 10.1074/jbc.273.41.26292

[58] Ferreon ACM , Moran CR , Ferreon JC & Deniz AA (2010) Alteration of the α‐synuclein folding landscape by a mutation related to Parkinson's disease. Angew Chem Int Ed 49, 3469–3472. 10.1002/anie.201000378 PMC2972640 20544898

[59] Seyfried TN , Choi H , Chevalier A , Hogan D , Akgoc Z & Schneider JS (2018) Sex‐related abnormalities in substantia nigra lipids in Parkinson's disease. ASN Neuro 10, 1759091418781889. 29932343 10.1177/1759091418781889 PMC6024349

[60] Li J , Uversky VN & Fink AL (2001) Effect of familial Parkinson's disease point mutations A30P and A53T on the structural properties, aggregation, and fibrillation of human α‐synuclein. Biochemistry 40, 11604–11613. 11560511 10.1021/bi010616g

[61] Van Meer G , Voelker DR & Feigenson GW (2008) Membrane lipids: where they are and how they behave. Nat Rev Mol Cell Biol 9, 112–124. 18216768 10.1038/nrm2330 PMC2642958

[62] Rudnik S & Damme M (2021) The lysosomal membrane—export of metabolites and beyond. FEBS J 288, 4168–4182. 33067905 10.1111/febs.15602

[63] Lv Z , Krasnoslobodtsev AV , Zhang Y , Ysselstein D , Rochet JC , Blanchard SC & Lyubchenko YL (2016) Effect of acidic pH on the stability of α‐synuclein dimers. Biopolymers 105, 715–724. 27177831 10.1002/bip.22874 PMC4958566

[64] Buell AK , Galvagnion C , Gaspar R , Sparr E , Vendruscolo M , Knowles TPJ , Linse S & Dobson CM (2014) Solution conditions determine the relative importance of nucleation and growth processes in α‐synuclein aggregation. Proc Natl Acad Sci USA 111, 7671–7676. 24817693 10.1073/pnas.1315346111 PMC4040554

[65] Jiang P , Gan M , Yen SH , McLean PJ & Dickson DW (2017) Impaired endo‐lysosomal membrane integrity accelerates the seeding progression of α‐synuclein aggregates. Sci Rep 7, 1–13. 28794446 10.1038/s41598-017-08149-w PMC5550496

[66] Blázquez L , Otaegui D , Sáenz A , Paisán‐Ruiz C , Emparanza JI , Ruiz‐Martinez J , Moreno F , Martí‐Massó JF & de Munain AL (2006) Apolipoprotein E epsilon4 allele in familial and sporadic Parkinson's disease. Neurosci Lett 406, 235–239. 16904828 10.1016/j.neulet.2006.07.037

[67] Factor SA , Steenland KN , Higgins DS , Molho ES , Kay DM , Montimurro J , Rosen AR , Zabetian CP & Payami H (2011) Postural instability/gait disturbance in Parkinson's disease has distinct subtypes: an exploratory analysis. J Neurol Neurosurg Psychiatry 82, 564–568. 20884673 10.1136/jnnp.2010.222042 PMC4646086

[68] Tsuang D , Leverenz JB , Lopez OL , Hamilton RL , Bennett DA , Schneider JA , Buchman AS , Larson EB , Crane PK , Kaye JA *et al*. (2013) APOE ɛ4 increases risk for dementia in pure synucleinopathies. JAMA Neurol 70, 223–228. 23407718 10.1001/jamaneurol.2013.600 PMC3580799

[69] Fernández‐Calle R , Konings SC , Frontiñán‐Rubio J , García‐Revilla J , Camprubí‐Ferrer L , Svensson M , Martinson I , Boza‐Serrano A , Venero JL , Nielsen HM *et al*. (2022) APOE in the bullseye of neurodegenerative diseases: impact of the APOE genotype in Alzheimer's disease pathology and brain diseases. Mol Neurodegener 17, 1–47. 36153580 10.1186/s13024-022-00566-4 PMC9509584

[70] de Leeuw SM , Kirschner AWT , Lindner K , Rust R , Budny V , Wolski WE , Gavin AC , Nitsch RM & Tackenberg C (2022) APOE2, E3, and E4 differentially modulate cellular homeostasis, cholesterol metabolism, and inflammatory response in isogenic iPSC‐derived astrocytes. Stem Cell Rep 17, 110–126. 10.1016/j.stemcr.2021.11.007 PMC8758949 34919811

[71] Boyles JK , Pitas RE , Wilson E , Mahley RW & Taylor JM (1985) Apolipoprotein E associated with astrocytic glia of the central nervous system and with nonmyelinating glia of the peripheral nervous system. J Clin Invest 76, 1501–1513. 3932467 10.1172/JCI112130 PMC424114

[72] Morikawa M , Fryer JD , Sullivan PM , Christopher EA , Wahrle SE , DeMattos RB , O'Dell MA , Fagan AM , Lashuel HA , Walz T *et al*. (2005) Production and characterization of astrocyte‐derived human apolipoprotein E isoforms from immortalized astrocytes and their interactions with amyloid‐β. Neurobiol Dis 19, 66–76. 15837562 10.1016/j.nbd.2004.11.005

[73] Dekroon RM & Armati PJ (2001) Synthesis and processing of apolipoprotein E in human brain cultures. Glia 33, 298–305. 11246228 10.1002/1098-1136(20010315)33:4<298::aid-glia1028>3.0.co;2-n

[74] Buttini M , Masliah E , Yu GQ , Palop JJ , Chang S , Bernardo A , Lin C , Wyss‐Coray T , Huang Y & Mucke L (2010) Cellular source of apolipoprotein E4 determines neuronal susceptibility to excitotoxic injury in transgenic mice. Am J Pathol 177, 563–569. 20595630 10.2353/ajpath.2010.090973 PMC2913361

[75] Xu Q , Bernardo A , Walker D , Kanegawa T , Mahley RW & Huang Y (2006) Profile and regulation of apolipoprotein E (ApoE) expression in the CNS in mice with targeting of green fluorescent protein gene to the ApoE locus. J Neurosci 26, 4985–4994. 16687490 10.1523/JNEUROSCI.5476-05.2006 PMC6674234

[76] Keren‐Shaul H , Spinrad A , Weiner A , Matcovitch‐Natan O , Dvir‐Szternfeld R , Ulland TK , David E , Baruch K , Lara‐Astaiso D , Toth B *et al*. (2017) A unique microglia type associated with restricting development of Alzheimer's disease. Cell 169, 1276–1290.e17. 28602351 10.1016/j.cell.2017.05.018

[77] Emamzadeh FN , Aojula H , McHugh PC & Allsop D (2016) Effects of different isoforms of apoE on aggregation of the α‐synuclein protein implicated in Parkinson's disease. Neurosci Lett 618, 146–151. 26921451 10.1016/j.neulet.2016.02.042

[78] Zhao J , Lu W , Ren Y , Fu Y , Martens YA , Shue F , Davis MD , Wang X , Chen K , Li F *et al*. (2021) Apolipoprotein E regulates lipid metabolism and α‐synuclein pathology in human iPSC‐derived cerebral organoids. Acta Neuropathol 142, 807–825. 34453582 10.1007/s00401-021-02361-9 PMC8500881

[79] Zhao N , Attrebi ON , Ren Y , Qiao W , Sonustun B , Martens YA , Meneses AD , Li F , Shue F , Zheng J *et al*. (2020) APOE4 exacerbates α‐synuclein pathology and related toxicity independent of amyloid. Sci Transl Med 12, 1809. 10.1126/scitranslmed.aay1809 PMC8309690 32024798

[80] Davis AA , Inman CE , Wargel ZM , Dube U , Freeberg BM , Galluppi A , Haines JN , Dhavale DD , Miller R , Choudhury FA *et al*. (2020) APOE genotype regulates pathology and disease progression in synucleinopathy. Sci Transl Med 12, 3069. 10.1126/scitranslmed.aay3069 PMC7289511 32024799

[81] Mann DMA , Pickering Brown SM , Owen F , Baba M & Iwatsubo T (1998) Amyloid β protein (Aβ) deposition in dementia with Lewy bodies: predominance of Aβ42(43) and paucity of Aβ40 compared with sporadic Alzheimer's disease. Neuropathol Appl Neurobiol 24, 187–194. 9717183 10.1046/j.1365-2990.1998.00112.x

[82] Dickson DW , Heckman MG , Murray ME , Soto AI , Walton RL , Diehl NN , Van Gerpen JA , Uitti RJ , Wszolek ZK , Ertekin‐Taner N *et al*. (2018) APOE 4 is associated with severity of Lewy body pathology independent of Alzheimer pathology. Neurology 91, E1182–E1195. 30143564 10.1212/WNL.0000000000006212 PMC6161556

[83] Troy TR & Jacob MM (2018) Apolipoprotein E fragmentation within Lewy bodies of the human Parkinson's disease brain. Int J Neurodegener Dis 1, 002. 30272057 10.23937/IJND-2017/1710002 PMC6159338

[84] Paslawski W , Zareba‐Paslawska J , Zhang X , Hölzl K , Wadensten H , Shariatgorji M , Janelidze S , Hansson O , Forsgren L , Andrén PE *et al*. (2019) α‐Synuclein‐lipoprotein interactions and elevated ApoE level in cerebrospinal fluid from Parkinson's disease patients. Proc Natl Acad Sci USA 116, 15226–15235. 31270237 10.1073/pnas.1821409116 PMC6660770

[85] Harhangi BS , De Rijk MC , Van Duijn CM , Van Broeckhoven C , Hofman A & Breteler MMB (2000) APOE and the risk of PD with or without dementia in a population‐based study. Neurology 54, 1272–1276. 10746597 10.1212/wnl.54.6.1272

[86] Federoff M , Jimenez‐Rolando B , Nalls MA & Singleton AB (2012) A large study reveals no association between APOE and Parkinson's disease. Neurobiol Dis 46, 389–392. 22349451 10.1016/j.nbd.2012.02.002 PMC3323723

[87] Mata IF , Leverenz JB , Weintraub D , Trojanowski JQ , Hurtig HI , Van Deerlin VM , Ritz B , Rausch R , Rhodes SL , Factor SA *et al*. (2014) APOE, MAPT, and SNCA genes and cognitive performance in Parkinson disease. JAMA Neurol 71, 1405–1412. 25178429 10.1001/jamaneurol.2014.1455 PMC4227942

[88] Tan MMX , Lawton MA , Jabbari E , Reynolds RH , Iwaki H , Blauwendraat C , Kanavou S , Pollard MI , Hubbard L , Malek N *et al*. (2021) Genome‐wide association studies of cognitive and motor progression in Parkinson's disease. Mov Disord 36, 424–433. 33111402 10.1002/mds.28342 PMC9053517

[89] Faraji P , Kühn H & Ahmadian S (2024) Multiple roles of apolipoprotein E4 in oxidative lipid metabolism and ferroptosis during the pathogenesis of Alzheimer's disease. J Mol Neurosci 74, 62. 38958788 10.1007/s12031-024-02224-4 PMC11222241

[90] Lozupone M & Panza F (2024) Impact of apolipoprotein E isoforms on sporadic Alzheimer's disease: beyond the role of amyloid beta. Neural Regen Res 19, 80–83. 37488848 10.4103/1673-5374.375316 PMC10479857

[91] Kloske CM , Belloy ME , Blue EE , Bowman GR , Carrillo MC , Chen X , Chiba‐Falek O , Davis AA , Di Paolo G , Garretti F *et al*. (2024) Advancements in APOE and dementia research: highlights from the 2023 AAIC advancements: APOE conference. Alzheimers Dement 20, 6590–6605. 39031528 10.1002/alz.13877 PMC11497726

[92] Krasemann S , Madore C , Cialic R , Baufeld C , Calcagno N , El Fatimy R , Beckers L , O'Loughlin E , Xu Y , Fanek Z *et al*. (2017) The TREM2‐APOE pathway drives the transcriptional phenotype of dysfunctional microglia in neurodegenerative diseases. Immunity 47, 566–581.e9. 28930663 10.1016/j.immuni.2017.08.008 PMC5719893

[93] Choi I , Zhang Y , Seegobin SP , Pruvost M , Wang Q , Purtell K , Zhang B & Yue Z (2020) Microglia clear neuron‐released α‐synuclein via selective autophagy and prevent neurodegeneration. Nat Commun 11, 1386. 32170061 10.1038/s41467-020-15119-w PMC7069981

[94] Reynolds NP , Soragni A , Rabe M , Verdes D , Liverani E , Handschin S , Riek R & Seeger S (2011) Mechanism of membrane interaction and disruption by α‐synuclein. J Am Chem Soc 133, 19366–19375. 21978222 10.1021/ja2029848

[95] Colebc NB , Murphy DD , Grider T , Rueter S , Brasaemle D & Nussbaum RL (2002) Lipid droplet binding and oligomerization properties of the Parkinson's disease protein α‐synuclein. J Biol Chem 277, 6344–6352. 11744721 10.1074/jbc.M108414200

[96] Cilia R , Tunesi S , Marotta G , Cereda E , Siri C , Tesei S , Zecchinelli AL , Canesi M , Mariani CB , Meucci N *et al*. (2016) Survival and dementia in GBA‐associated Parkinson's disease: the mutation matters. Ann Neurol 80, 662–673. 27632223 10.1002/ana.24777

[97] Parlar SC , Grenn FP , Kim JJ , Baluwendraat C & Gan‐Or Z (2023) Classification of GBA1 variants in Parkinson's disease: the GBA1‐PD browser. Mov Disord 38, 489–495. 36598340 10.1002/mds.29314 PMC10033371

[98] Sidransky E , Nalls MA , Aasly JO , Aharon‐Peretz J , Annesi G , Barbosa ER , Bar‐Shira A , Berg D , Bras J , Brice A *et al*. (2009) Multicenter analysis of glucocerebrosidase mutations in Parkinson's disease. N Engl J Med 361, 1651–1661. 19846850 10.1056/NEJMoa0901281 PMC2856322

[99] Guerreiro R , Ross OA , Kun‐Rodrigues C , Hernandez DG , Orme T , Eicher JD , Shepherd CE , Parkkinen L , Darwent L , Heckman MG *et al*. (2018) Investigating the genetic architecture of dementia with Lewy bodies: a two‐stage genome‐wide association study. Lancet Neurol 17, 64–74. 29263008 10.1016/S1474-4422(17)30400-3 PMC5805394

[100] Rongve A , Witoelar A , Ruiz A , Athanasiu L , Abdelnour C , Clarimon J , Heilmann‐Heimbach S , Hernández I , Moreno‐Grau S , de Rojas I *et al*. (2019) GBA and APOE ε4 associate with sporadic dementia with Lewy bodies in European genome wide association study. Sci Rep 9, 7013. 31065058 10.1038/s41598-019-43458-2 PMC6504850

[101] Gegg ME , Menozzi E & Schapira AHV (2022) Glucocerebrosidase‐associated Parkinson disease: pathogenic mechanisms and potential drug treatments. Neurobiol Dis 166, 105663. 35183702 10.1016/j.nbd.2022.105663

[102] Senkevich K , Rudakou U & Gan‐Or Z (2023) Genetic mechanism vs genetic subtypes: the example of GBA. Handb Clin Neurol 193, 155–170. 36803808 10.1016/B978-0-323-85555-6.00016-3

[103] Ron I & Horowitz M (2005) ER retention and degradation as the molecular basis underlying Gaucher disease heterogeneity. Hum Mol Genet 14, 2387–2398. 16000318 10.1093/hmg/ddi240

[104] Bendikov‐Bar I , Ron I , Filocamo M & Horowitz M (2011) Characterization of the ERAD process of the L444P mutant glucocerebrosidase variant. Blood Cells Mol Dis 46, 4–10. 21106416 10.1016/j.bcmd.2010.10.012

[105] McNeill A , Magalhaes J , Shen C , Chau KY , Hughes D , Mehta A , Foltynie T , Cooper JM , Abramov AY , Gegg M *et al*. (2014) Ambroxol improves lysosomal biochemistry in glucocerebrosidase mutation‐linked Parkinson disease cells. Brain 137, 1481–1495. 24574503 10.1093/brain/awu020 PMC3999713

[106] Di Domenico F & Lanzillotta C (2022) The disturbance of protein synthesis/degradation homeostasis is a common trait of age‐related neurodegenerative disorders. Adv Protein Chem Struct Biol 132, 49–87. 36088079 10.1016/bs.apcsb.2022.05.008

[107] Da Costa CA , El Manaa W , Duplan E & Checler F (2020) The endoplasmic reticulum stress/unfolded protein response and their contributions to Parkinson's disease physiopathology. Cells 9, 2495. 33212954 10.3390/cells9112495 PMC7698446

[108] Parenti G , Andria G & Valenzano KJ (2015) Pharmacological chaperone therapy: preclinical development, clinical translation, and prospects for the treatment of lysosomal storage disorders. Mol Ther 23, 1138–1148. 25881001 10.1038/mt.2015.62 PMC4817787

[109] Kuo SH , Tasset I , Cheng MM , Diaz A , Pan MK , Lieberman OJ , Hutten SJ , Alcalay RN , Kim S , Ximénez‐Embún P *et al*. (2022) Mutant glucocerebrosidase impairs α‐synuclein degradation by blockade of chaperone‐mediated autophagy. Sci Adv 8, 6393. 10.1126/sciadv.abm6393 PMC11809618 35138901

[110] Fernandes HJR , Hartfield EM , Christian HC , Emmanoulidou E , Zheng Y , Booth H , Bogetofte H , Lang C , Ryan BJ , Sardi SP *et al*. (2016) ER stress and autophagic perturbations lead to elevated extracellular α‐synuclein in GBA‐N370S Parkinson's iPSC‐derived dopamine neurons. Stem Cell Rep 6, 342–356. 10.1016/j.stemcr.2016.01.013 PMC4788783 26905200

[111] Avisar H , Guardia‐Laguarta C , Area‐Gomez E , Surface M , Chan AK , Alcalay RN & Lerner B (2021) Lipidomics prediction of Parkinson's disease severity: a machine‐learning analysis. J Parkinsons Dis 11, 1141–1155. 33814463 10.3233/JPD-202476 PMC8355022

[112] Guedes LC , Chan RB , Gomes MA , Conceição VA , Machado RB , Soares T , Xu Y , Gaspar P , Carriço JA , Alcalay RN *et al*. (2017) Serum lipid alterations in GBA‐associated Parkinson's disease. Parkinsonism Relat Disord 44, 58–65. 28890071 10.1016/j.parkreldis.2017.08.026

[113] Huebecker M , Moloney EB , Van Der Spoel AC , Priestman DA , Isacson O , Hallett PJ & Platt FM (2019) Reduced sphingolipid hydrolase activities, substrate accumulation and ganglioside decline in Parkinson's disease. Mol Neurodegener 14, 1–21. 31703585 10.1186/s13024-019-0339-z PMC6842240

[114] Orme T , Guerreiro R & Bras J (2018) The genetics of dementia with Lewy bodies: current understanding and future directions. Curr Neurol Neurosci Rep 18, 1–13. 30097731 10.1007/s11910-018-0874-y PMC6097049

[115] Goddard TR , Brookes KJ , Sharma R , Moemeni A & Rajkumar AP (2024) Dementia with Lewy bodies: genomics, transcriptomics, and its future with data science. Cells 13, 223. 38334615 10.3390/cells13030223 PMC10854541

[116] Mazzulli JR , Xu YH , Sun Y , Knight AL , McLean PJ , Caldwell GA , Sidransky E , Grabowski GA & Krainc D (2011) Gaucher disease glucocerebrosidase and α‐synuclein form a bidirectional pathogenic loop in synucleinopathies. Cell 146, 37–52. 21700325 10.1016/j.cell.2011.06.001 PMC3132082

[117] Fagone P & Jackowski S (2009) Membrane phospholipid synthesis and endoplasmic reticulum function. J Lipid Res 50, S311–S316. 18952570 10.1194/jlr.R800049-JLR200 PMC2674712

[118] Wang S , Chen Z , Lam V , Han J , Hassler J , Finck BN , Davidson NO & Kaufman RJ (2012) IRE1α‐XBP1s induces PDI expression to increase MTP activity for hepatic VLDL assembly and lipid homeostasis. Cell Metab 16, 473–486. 23040069 10.1016/j.cmet.2012.09.003 PMC3569089

[119] Lee AH , Scapa EF , Cohen DE & Glimcher LH (2008) Regulation of hepatic lipogenesis by the transcription factor XBP1. Science 320, 1492–1496. 18556558 10.1126/science.1158042 PMC3620093

[120] Lauressergues E , Bert E , Duriez P , Hum D , Majd Z , Staels B & Cussac D (2012) Does endoplasmic reticulum stress participate in APD‐induced hepatic metabolic dysregulation? Neuropharmacology 62, 784–796. 21924277 10.1016/j.neuropharm.2011.08.048

[121] Li H , Meng Q , Xiao F , Chen S , Du Y , Yu J , Wang C & Guo F (2011) ATF4 deficiency protects mice from high‐carbohydrate‐diet‐induced liver steatosis. Biochem J 438, 283–289. 21644928 10.1042/BJ20110263

[122] Ho N , Xu C & Thibault G (2018) From the unfolded protein response to metabolic diseases – lipids under the spotlight. J Cell Sci 131, jcs199307. 29439157 10.1242/jcs.199307

[123] Ioannou MS , Jackson J , Sheu SH , Chang CL , Weigel AV , Liu H , Pasolli HA , Xu CS , Pang S , Matthies D *et al*. (2019) Neuron‐astrocyte metabolic coupling protects against activity‐induced fatty acid toxicity. Cell 177, 1522–1535.e14. 31130380 10.1016/j.cell.2019.04.001

[124] Isacson O , Brekk OR & Hallett PJ (2019) Novel results and concepts emerging from lipid cell biology relevant to degenerative brain aging and disease. Front Neurol 10, 479126. 10.3389/fneur.2019.01053 PMC6794469 31649605

[125] Brekk OR , Honey JR , Lee S , Hallett PJ & Isacson O (2020) Cell type‐specific lipid storage changes in Parkinson's disease patient brains are recapitulated by experimental glycolipid disturbance. Proc Natl Acad Sci USA 117, 27646–27654. 33060302 10.1073/pnas.2003021117 PMC7959493

[126] Szwedo AA , Dalen I , Pedersen KF , Camacho M , Bäckström D , Forsgren L , Tzoulis C , Winder‐Rhodes S , Hudson G , Liu G *et al*. (2022) GBA and APOE impact cognitive decline in Parkinson's disease: a 10‐year population‐based study. Mov Disord 37, 1016–1027. 35106798 10.1002/mds.28932 PMC9362732

[127] Connolly KJ , Margaria J , Di Biase E , Cooper O , Hallett PJ & Isacson O (2023) Loss of lipid carrier ApoE exacerbates brain glial and inflammatory responses after lysosomal GBA1 inhibition. Cells 12, 2564. 37947642 10.3390/cells12212564 PMC10647680

[128] Frasier M , Fiske BK & Sherer TB (2022) Precision medicine for Parkinson's disease: the subtyping challenge. Front Aging Neurosci 14, 1064057. 36533178 10.3389/fnagi.2022.1064057 PMC9751632

[129] Stige KE , Kverneng SU , Sharma S , Skeie GO , Sheard E , Søgnen M , Geijerstam SA , Vetås T , Wahlvåg AG , Berven H *et al*. (2024) The STRAT‐PARK cohort: a personalized initiative to stratify Parkinson's disease. Prog Neurobiol 236, 102603. 38604582 10.1016/j.pneurobio.2024.102603

[130] Outeiro TF , Kalia LV , Bezard E , Ferrario J , Lin CH , Salama M , Standaert DG , Taiwo L , Takahashi R , Vila M *et al*. (2024) Basic science in movement disorders: fueling the engine of translation into clinical practice. Mov Disord 39, 929–933. 38576081 10.1002/mds.29802

[131] Wik L , Nordberg N , Broberg J , Björkesten J , Assarsson E , Henriksson S , Grundberg I , Pettersson E , Westerberg C , Liljeroth E *et al*. (2021) Proximity extension assay in combination with next‐generation sequencing for high‐throughput proteome‐wide analysis. Mol Cell Proteomics 20, 100168. 34715355 10.1016/j.mcpro.2021.100168 PMC8633680

[132] Cilento EM , Jin L , Stewart T , Shi M , Sheng L & Zhang J (2019) Mass spectrometry: a platform for biomarker discovery and validation for Alzheimer's and Parkinson's diseases. J Neurochem 151, 397–416. 30474862 10.1111/jnc.14635 PMC6535381

[133] Shao Y , Li T , Liu Z , Wang X , Xu X , Li S , Xu G & Le W (2021) Comprehensive metabolic profiling of Parkinson's disease by liquid chromatography‐mass spectrometry. Mol Neurodegener 16, 1–15. 33485385 10.1186/s13024-021-00425-8 PMC7825156

[134] Hällqvist J , Bartl M , Dakna M , Schade S , Garagnani P , Bacalini MG , Pirazzini C , Bhatia K , Schreglmann S , Xylaki M *et al*. (2024) Plasma proteomics identify biomarkers predicting Parkinson's disease up to 7 years before symptom onset. Nat Commun 15, 4759. 38890280 10.1038/s41467-024-48961-3 PMC11189460

[135] Maple‐Grødem J , Ushakova A , Pedersen KF , Tysnes OB , Alves G & Lange J (2023) Identification of diagnostic and prognostic biomarkers of PD using a multiplex proteomics approach. Neurobiol Dis 186, 106281. 37673381 10.1016/j.nbd.2023.106281

[136] Feleke R , Reynolds RH , Smith AM , Tilley B , Taliun SAG , Hardy J , Matthews PM , Gentleman S , Owen DR , Johnson MR *et al*. (2021) Cross‐platform transcriptional profiling identifies common and distinct molecular pathologies in Lewy body diseases. Acta Neuropathol 142, 449–474. 34309761 10.1007/s00401-021-02343-x PMC8357687

[137] Salemi M , Lanza G , Mogavero MP , Cosentino FII , Borgione E , Iorio R , Ventola GM , Marchese G , Salluzzo MG , Ravo M *et al*. (2022) A transcriptome analysis of mRNAs and long non‐coding RNAs in patients with Parkinson's disease. Int J Mol Sci 23, 1535. 35163455 10.3390/ijms23031535 PMC8836138

[138] Marques‐Coelho D , Lohan LCC , Melo de Farias AR , Flaig A , Letournel F , Martin‐Négrier ML , Chapon F , Faisant M , Godfraind C , Maurage CA *et al*. (2021) Differential transcript usage unravels gene expression alterations in Alzheimer's disease human brains. NPJ Aging Mech Dis 7, 1–15. 33398016 10.1038/s41514-020-00052-5 PMC7782705

[139] Wu H , Wang J , Hu X , Zhuang C , Zhou J , Wu P , Li S & Zhao RC (2023) Comprehensive transcript‐level analysis reveals transcriptional reprogramming during the progression of Alzheimer's disease. Front Aging Neurosci 15, 1191680. 37396652 10.3389/fnagi.2023.1191680 PMC10308376

[140] Lewis CM & Vassos E (2020) Polygenic risk scores: from research tools to clinical instruments. Genome Med 12, 44. 32423490 10.1186/s13073-020-00742-5 PMC7236300

[141] Choi SW , Mak TSH & O'Reilly PF (2020) Tutorial: a guide to performing polygenic risk score analyses. Nat Protoc 15, 2759–2772. 32709988 10.1038/s41596-020-0353-1 PMC7612115

[142] Wright LG , Onodera T , Stein MM , Wang T , Schachter DT , Hu Z & McMahon PL (2022) Deep physical neural networks trained with backpropagation. Nature 601, 549–555. 35082422 10.1038/s41586-021-04223-6 PMC8791835

[143] Badré A , Zhang L , Muchero W , Reynolds JC & Pan C (2020) Deep neural network improves the estimation of polygenic risk scores for breast cancer. J Hum Genet 66, 359–369. 33009504 10.1038/s10038-020-00832-7

[144] Castellanos DB , Martín‐Jiménez CA , Rojas‐Rodríguez F , Barreto GE & González J (2021) Brain lipidomics as a rising field in neurodegenerative contexts: perspectives with machine learning approaches. Front Neuroendocrinol 61, 100899. 33450200 10.1016/j.yfrne.2021.100899

[145] Wang R , Li B , Lam SM & Shui G (2020) Integration of lipidomics and metabolomics for in‐depth understanding of cellular mechanism and disease progression. J Genet Genomics 47, 69–83. 32178981 10.1016/j.jgg.2019.11.009

[146] Cheng D , Jenner AM , Shui G , Cheong WF , Mitchell TW , Nealon JR , Kim WS , McCann H , Wenk MR , Halliday GM *et al*. (2011) Lipid pathway alterations in Parkinson's disease primary visual cortex. PLoS One 6, e17299. 21387008 10.1371/journal.pone.0017299 PMC3046155

[147] Marin R , Fabelo N , Martín V , Garcia‐Esparcia P , Ferrer I , Quinto‐Alemany D & Díaz M (2017) Anomalies occurring in lipid profiles and protein distribution in frontal cortex lipid rafts in dementia with Lewy bodies disclose neurochemical traits partially shared by Alzheimer's and Parkinson's diseases. Neurobiol Aging 49, 52–59. 27768960 10.1016/j.neurobiolaging.2016.08.027

[148] Guo X , Song W , Chen K , Chen XP , Zheng Z , Cao B , Huang R , Zhao B , Wu Y & Shang HF (2015) The serum lipid profile of Parkinson's disease patients: a study from China. Int J Neurosci 125, 838–844. 25340257 10.3109/00207454.2014.979288

[149] Xicoy H , Brouwers JF , Wieringa B & Martens GJM (2020) Explorative combined lipid and transcriptomic profiling of substantia nigra and putamen in Parkinson's disease. Cells 9, 1966. 32858884 10.3390/cells9091966 PMC7564986

[150] Mielke MM , Maetzler W , Haughey NJ , Bandaru VVR , Savica R , Deuschle C , Gasser T , Hauser AK , Gräber‐Sultan S , Schleicher E *et al*. (2013) Plasma ceramide and glucosylceramide metabolism is altered in sporadic Parkinson's disease and associated with cognitive impairment: a pilot study. PLoS One 8, e73094. 24058461 10.1371/journal.pone.0073094 PMC3776817

[151] Huh YE , Park H , Chiang MSR , Tuncali I , Liu G , Locascio JJ , Shirvan J , Hutten SJ , Rotunno MS , Viel C *et al*. (2021) Glucosylceramide in cerebrospinal fluid of patients with GBA‐associated and idiopathic Parkinson's disease enrolled in PPMI. NPJ Parkins Dis 7, 102. 10.1038/s41531-021-00241-3 PMC8608962 34811369

[152] Lv J , Zhang L , Yan F & Wang X (2018) Clinical lipidomics: a new way to diagnose human diseases. Clin Transl Med 7, e12. 10.1186/s40169-018-0190-9 PMC5923182 29704148

